# Tunable Surfactant-Assisted WO_3_ Nanogranules as High-Performance Electrocatalysts for the Oxygen Evolution Reaction

**DOI:** 10.3390/ma18092129

**Published:** 2025-05-06

**Authors:** Mrunal Bhosale, Pritam J. Morankar, Rutuja U. Amate, Chan-Wook Jeon

**Affiliations:** School of Chemical Engineering, Yeungnam University, 280 Daehak-ro, Gyeongsan 712-749, Republic of Korea; mrunal.snst.1@gmail.com (M.B.); pritam.nanoworld@gmail.com (P.J.M.); rutu.nanoworld@gmail.com (R.U.A.)

**Keywords:** oxygen evolution reaction, water splitting: electrocatalyst, PEG-WO_3_, electrochemical stability

## Abstract

Addressing the global energy demand requires the development of sustainable and highly efficient technologies for clean energy generation. One of the primary challenges in the oxygen evolution reaction (OER) is overcoming sluggish reaction kinetics, which requires the design of electrocatalysts with greater activity and long-term stability. In this study, a precipitation method was employed to synthesize polyethylene glycol (PEG) assisted tungsten oxide (WO_3_) as an effective and stable electrocatalyst for OER. PEG was incorporated at varying concentrations (1%, 3%, and 5%) to modulate the structural and electrochemical characteristics of WO_3_. Among the resulting composites, the sample with 3% PEG (PEG-WO_3_-2) exhibited the most favorable catalytic behavior, achieving a low overpotential of 407.7 mV at a current density of 10 mA cm^−2^ and a Tafel slope of 76.2 mV dec^−1^ in 1 M KOH electrolyte. Furthermore, long-term electrochemical stability was evaluated over 5000 consecutive cycles, revealing minimal degradation in catalytic activity. The heightened performance is attributed to the optimized composition, improved electron transport properties, and the presence of a higher density of active sites, all of which contribute to the superior catalytic activity of the PEG-WO_3_-2 electrocatalyst.

## 1. Introduction

Hydrogen is widely recognized as a clean and sustainable energy vector owing to its zero-carbon footprint and exceptionally high energy density (approximately 142 MJ kg^−1^), making it a promising candidate for future green energy systems [1]. Among the various hydrogen production strategies, electrochemical water splitting has garnered significant attention due to its capability to generate hydrogen using renewable energy inputs, thereby supporting the development of a sustainable hydrogen economy [2,3]. This process comprises two half-cell reactions: the hydrogen evolution reaction (HER) occurring at the cathode and the oxygen evolution reaction (OER) at the anode [4]. However, this technique is highly dependent on the selectivity, activity, and stability of the electrocatalyst [5,6,7]. Despite its potential, the overall efficiency of water splitting is severely constrained by the OER, especially under alkaline conditions. This is primarily attributed to its intrinsically sluggish kinetics, which stem from a complex, multi-step electron transfer pathway involving the transfer of four electrons [8,9]. As a result, a significant overpotential is required to drive the reaction at practical rates, thereby rendering the OER the rate-limiting step in the water splitting process [10,11]. To address these limitations, recent research efforts have focused on the rational design of advanced electrocatalysts, particularly multi-component systems with large electrochemically active surface areas (ECSA). These engineered materials aim to enhance intrinsic activity, accelerate charge transfer, and improve long-term durability, collectively contributing to more efficient OER performance under alkaline conditions [12,13].

The design of OER electrocatalysts is primarily guided by the need to achieve high intrinsic catalytic activity, characterized by low overpotential, while maintaining structural and electrochemical stability over prolonged operational periods. Transition metal-based electrocatalysts exhibit tunable electronic structure and high atomic utilization, which is beneficial for efficient electrochemical performance [14]. Despite significant advancements, many state-of-the-art oxide systems, such as spinel-type oxides, perovskite-like oxides, and Co-, Mn-, or Fe-based binary oxides, still exhibit high overpotentials, poor corrosion resistance, and limited long-term stability [6,15,16,17,18]. For instance, many Fe- and Co-based oxides suffer from low intrinsic conductivity and require additional conductive supports, while perovskite electrocatalysts often undergo structural degradation under operating conditions [18,19]. Ji et al. developed Ca-doped CuCoO_2_ nanosheets via a surfactant-assisted hydrothermal synthesis incorporating polyvinylpyrrolidone (PVP), which demonstrated an improved OER performance with an overpotential of 470 mV at a current density of 10 mA cm^−2^ [20]. Miao et al. reported the fabrication of Ni-doped CuCoO_2_ (CCO) derived from Cu-BTC through a solvothermal route, where the optimized Ni@5NCCO catalyst required only 409 mV to achieve the same benchmark current density [21]. In another study, Graeme et al. synthesized cubic-phase LiCoO_2,_ which exhibited promising electrocatalytic activity for the OER, delivering an overpotential of approximately 420 mV at 10 mA cm^−2^ [22]. Additionally, Jie et al. engineered a three-dimensional ordered macroporous (3DOM) architecture of LaFeO_3_ perovskite, resulting in a significant enhancement in bifunctional activity for both OER and HER. The 3DOM-LaFeO_3_ catalyst achieved an overpotential of 420 mV at 10 mA cm^−2^ [23]. Tungsten trioxide (WO_3_), a wide-bandgap semiconductor with excellent chemical and thermal stability, has recently garnered interest as a potential catalyst or catalyst support due to its advantageous properties, such as environmental friendliness, natural abundance, high oxygen vacancy tunability, non-toxicity, and chemical resilience across a broad pH spectrum. These features render WO_3_ suitable for diverse electrochemical applications in both liquid-phase and gas-phase environments [24,25,26,27]. Notably, WO_3_ possesses a high oxygen storage capacity and can undergo reversible oxygen ion exchange at its surface, which enhances its redox behavior and interfacial activity [25,26]. These characteristics have led to its integration in a variety of energy-related technologies, including water splitting, photocatalysis, and gas sensing platforms. At the same time, WO_3_ has demonstrated commendable performance in HER. However, its role in OER catalysis remains underexplored, particularly as an individual or hybrid catalyst in alkaline media. This indicates a knowledge gap that merits further investigation into tungsten oxide and its derivatives as potential electrocatalysts for the OER, particularly through the application of established and facile synthesis strategies. The structural and morphological properties of nanomaterials are critically influenced by various synthesis parameters, among which the choice of surfactant plays a pivotal role. Polyethylene glycol (PEG), a widely utilized non-ionic surfactant, has demonstrated considerable efficacy in directing nanoparticle formation. Its presence during synthesis can modulate nucleation and growth dynamics, effectively controlling particle size and shape. PEG functions as a steric stabilizer and dispersing agent, preventing particle agglomeration by suppressing rapid crystallization and promoting uniform distribution through controlled kinetics. This regulation enables the formation of well-defined nanostructures with enhanced surface characteristics, which are highly desirable for catalytic applications [28,29].

This study systematically explores the influence of crystalline phase evolution on the electrocatalytic behavior of PEG-assisted WO_3_ nanostructures for the OER. By optimizing the ratio of PEG during synthesis, nanogranular WO_3_ composites were successfully fabricated, exhibiting tailored morphology and structural integrity. Comprehensive characterization techniques, including X-ray diffraction (XRD), Raman spectroscopy, X-ray photoelectron spectroscopy (XPS), and scanning electron microscopy (SEM), were employed to investigate the crystallographic features, surface chemistry, and microstructural attributes of the synthesized catalysts. To further understand the surface-related catalytic mechanisms, the electrochemically active surface area (ECSA) was quantitatively assessed. The optimized composition designated as PEG-WO_3_-2 demonstrated superior OER performance, delivering a low overpotential of 407.7 mV at 10 mA cm^−2^ and a Tafel slope of 76.2 mV dec^−1,^ indicative of accelerated reaction kinetics and efficient charge transfer. These results affirm that the synergistic effects induced by PEG incorporation significantly enhance the electrocatalytic properties of WO_3,_ positioning it as a promising candidate for high-performance OER applications.

## 2. Experimental Section

### 2.1. Chemicals

Hydrochloric acid (HCl, extra pure) and ethanol (EtOH, 94.5%) were acquired from Duksan Chemicals, Seoul, Republic of Korea. Potassium hydroxide (KOH, purity > 85%) was supplied by DaeJung Chemicals & Metals, Gyeonggi-do, Republic of Korea. Acetylene black (purity ≥ 99.9%) was obtained from Thermo Scientific, Seoul, Republic of Korea. Sodium tungstate dihydrate (Na_2_WO_4_·2H_2_O, ≥99% purity), poly(ethylene glycol) (PEG), polyvinylidene fluoride (PVDF), and N-methyl-2-pyrrolidone (NMP, ≥99% purity) were sourced from Sigma–Aldrich, St. Louis, MO, USA. The carbon cloth utilized in this study was procured from NARA Cell-Tech Corporation, Seoul, Republic of Korea. All chemicals were employed without further purification, and deionized (DI) water was used exclusively in all experimental procedures.

### 2.2. Preparation of Pure and WO_3_ and PEG Assisted WO_3_

**Figure 1:**
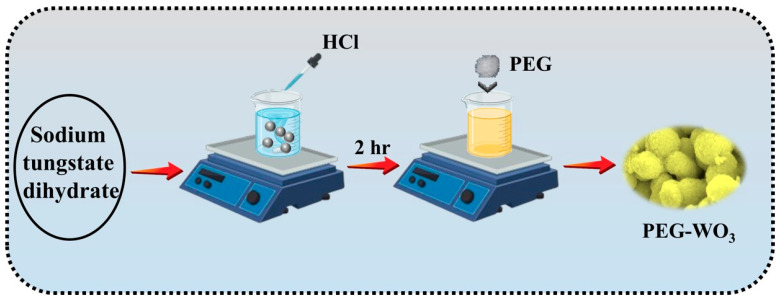
Schematic illustration of the PEG-WO_3_ electrocatalysts synthesis process.

The synthesis of WO_3_ nanogranular structures was performed using a hydrochloric acid-assisted precipitation method illustrated in Figure 1. The precursor solution was prepared by dissolving sodium tungstate dihydrate in 50 mL of DI water, followed by continuous stirring for 30 min to ensure complete dissolution. Subsequently, diluted HCl was added dropwise to the homogeneous solution until the pH was adjusted to approximately 2. The solution was then stirred vigorously for an additional 2 h and left undisturbed overnight to allow for precipitation. The supernatant was carefully removed, and the resulting precipitate was thoroughly washed and dried at 120 °C for 3 h. The dried sample was further subjected to annealing at 450 °C for 3 h and subsequently ground using an agate mortar and pestle. The synthesis of PEG-assisted WO_3_ followed the same procedure with the incorporation of PEG at concentrations of 1%, 3%, and 5% into the WO_3_ matrix. The resulting samples were designated as PEG-WO_3_-1, PEG-WO_3_-2, and PEG-WO_3_-3, respectively, to investigate their effect on the materials’ structural and catalytic properties.

### 2.3. Material Characterization

The structural characteristics and phase purity of the synthesized nanomaterials were analyzed using an X-ray diffractometer (X’Pert Pro) (Malvern Panalytical Ltd., Almelo, The Netherlands) equipped with a Cu Kα radiation source. Raman spectroscopy was conducted using an XploRA Plus system (HORIBA Jobin Yvon S.A.S, Paris, France) to investigate vibrational properties. X-ray photoelectron spectroscopy (XPS) was performed using a Thermo Scientific K-Alpha surface analysis system (Thermo Fisher Scientific Inc., Cheshire, UK) to examine the surface chemical composition and oxidation states. The surface morphology, particle size distribution, elemental composition, and elemental mapping were characterized using scanning electron microscopy (SEM, HITACHI S-4800, Hitachi, Tokyo, Japan) equipped with an energy-dispersive X-ray analysis (EDAX) system.

### 2.4. Electrochemical Analysis

All the electrochemical measurements were performed using a Biologic Instrument-WBCS3000 battery cycler (BioLogic, Gières, France). A conventional three-electrode arrangement was employed for comprehensive analysis. Prior to coating the electrocatalyst onto carbon cloth (CC), the substrate underwent a pretreatment process involving sequential immersion in 1 M HCl, DI water, and ethanol for 20 min each, followed by drying at 70 °C overnight. The electrocatalyst paste was formulated with an 80:10:10 weight ratio of active material, PVDF, and acetylene black using NMP as the solvent. The prepared slurry was uniformly coated onto a pretreated 1 × 1 cm^2^ carbon cloth and dried at 60 °C overnight to ensure proper adhesion. For electrochemical characterization, the coated carbon cloth functioned as the working electrode, while the Hg/HgO electrode and a platinum plate functioned as the reference and counter electrodes, respectively. All electrochemical measurements were carried out in a N_2_-saturated 1 M KOH electrolyte solution. Cyclic voltammetry (CV) was analyzed in the non-Faradaic region within a potential range of 0.1 to 0.2 V at varying scan rates of 5, 10, 15, 20, and 25 mV s^−1^. The ECSA was subsequently calculated using the following equation [30]:

(1) $$ECSA=\frac{C_{dl}}{C_{s}}$$

Here, C_dl_ signifies the electrochemical double-layer capacitance, while Cs indicates the specific capacitance of a planar surface in a 1 M KOH electrolyte, which is reported to be 0.040 mF cm^−2^ [31,32]. Electrochemical impedance spectroscopy (EIS) was performed on all samples using a 10 mV amplitude within a frequency range from 100 kHz to 0.1 Hz to evaluate charge transfer resistance and interfacial properties. To determine the overpotential for the OER linear sweep voltammetry (LSV) was conducted at a scan rate of 5 mV s^−1^ within a potential range of 0 to 1 V. All electrochemical measurements were performed using Hg/HgO reference electrode and the obtained data were converted to the reversible hydrogen electrode (RHE) scale using the Nernst equation as expressed below.

E_RHE_ = E_Hg/HgO_ + E°_Hg/HgO_ + 0.0591 × (pH) (2)

In this context, the pH of a 1 M KOH solution is approximately 13.9, and E°_Hg/HgO_ represents the standard electrode potential of the Hg/HgO reference electrode.

Furthermore, the Tafel slopes were determined by fitting the linear region of the Tafel plot to evaluate the reaction kinetics. The overpotential (η) for the oxygen evolution reaction was determined by deducting the standard potential for water oxidation from the experimentally obtained potential values using the following equation.

η = E (vs. RHE) − 1.23 V(3)

LSV was utilized to relate the optimized electrocatalysts’ stability before and after 5000 cycles of the CV study.

## 3. Results and Discussion

**Figure 2:**
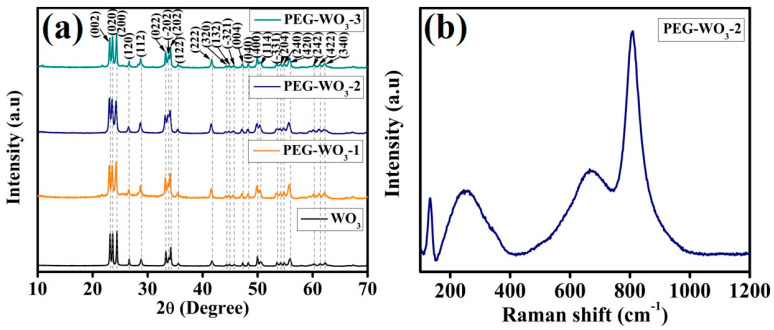
(**a**) XRD spectra of all the electrocatalysts and (**b**) Raman spectra of PEG-WO_3_-2 electrocatalyst.

X-ray diffraction (XRD) analysis was performed to assess the phase purity, crystal structure, and degree of crystallinity of the synthesized materials. Figure 2a illustrates the XRD pattern of WO_3_, PEG-WO_3_-1, PEG-WO_3_-2, and PEG-WO_3_-3 materials. The pristine WO_3_ exhibit the diffraction peaks at 23.1°, 23.6°, 24.4°, 26.6°, 28.8°, 33.3°, 33.9°, 34.2°, 35.5°, 41.7°, 44.3°, 44.8°, 45.7°, 47.2°, 48.3°, 49.9°, 50.5°, 53.5°, 54.2°, 54.8°, 55.9°, 60.2°, 61.4°, 62.2°. All the peaks are corresponding to the (002), (020), (200), (120), (112), (022), (–202), (202), (122), (222), (320), (132), (–321), (004), (040), (400), (114), (–331), (204), (240), (420), (242), (422), (340) lattice planes. The WO_3_ diffraction peaks provide the confirmation of a monoclinic structure, which is well matched with the JCPDS No. 01-083-0950 [33]. The absence of any additional diffraction peaks confirms the formation of a single-phase material. The surfactant-assisted WO_3_ samples exhibited diffraction patterns similar to those of pure WO_3_ with a slight shift toward lower angles. This shift indicates the presence of a more compact structural arrangement in the PEG-WO_3_-1, PEG-WO_3_-2, and PEG-WO_3_-3 samples, likely due to the influence of PEG in modulating the crystal lattice. Raman spectroscopy was conducted to analyze the molecular structure of the synthesized materials and their composite, as presented in Figure 2b. The spectra exhibit two distinct sets of peaks within the ranges of 100–400 and 600–900 cm^−1^. These peaks correspond to the bending and stretching vibrational modes of W=O and W-O-W bonds, respectively. The PEG-WO_3_ nanocomposites exhibit the Raman scattering peaks at 133.3 and 256.9 cm^−1^ from antisymmetric stretching and bending vibration modes of O-W-O bonds [33,34,35,36]. Furthermore, a peak observed at 665.3 cm^−1^ is revealing W-O stretching vibrations in the O-W-O chains of WO_3_ [37]. Additionally, the prominent peak exhibits the stretching vibrations of the bridging O-W-O bond, resulting in a peak corresponding to 810 cm^−1^ [33,38].

**Figure 3:**
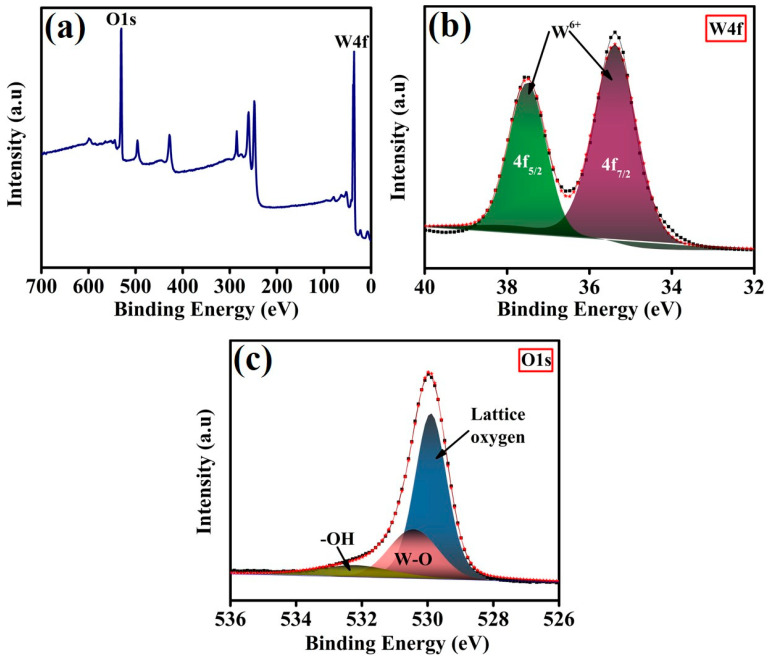
(**a**) XPS survey spectra and high-resolution XPS spectra of (**b**) W4f and (**c**) O1s for PEG-WO_3_-2 electrocatalyst.

The XPS characterization technique was employed to analyze the surface states and elemental composition of the developed materials. The obtained XPS results for the PEG-WO_3_-2 electrocatalyst are illustrated in Figure 3. The XPS survey spectrum (Figure 3a) reveals the characteristic peaks corresponding to W4f and O1s, confirming the presence of tungsten and oxygen in the PEG-WO_3_-2 material. To gain further insight into the chemical states of the elements, the deconvolution of the high-resolution spectra of W4f and O1s is presented in Figure 3b and Figure 3c, respectively. The deconvoluted W4f spectrum (Figure 3b) displays two distinctive peaks at binding energies of 35.2 eV and 37.3 eV. The peak at 35.2 eV corresponds to the 4f_7/2_ orbital, whereas the peak at 37.3 eV is attributed to the 4f_5/2_ orbital of W^6+^ in the monoclinic WO_3_ phase [39,40,41]. Figure 3c displays the O1S spectra exhibited in PEG-WO_3_-2, demonstrating that the peak located at 529.8 eV is associated with the lattice oxygen anions [42,43]. Additionally, two more peaks, deconvoluted at 530.4 and 532.3 eV, are assigned to the W-O bond in WO_3_ and surface hydroxide species [40,44]. Prominent chemical states are favorable to the adsorption of active species and contribute to the charge transfer during the rate-limiting steps, thereby facilitating the outstanding electrochemical performance.

**Figure 4:**
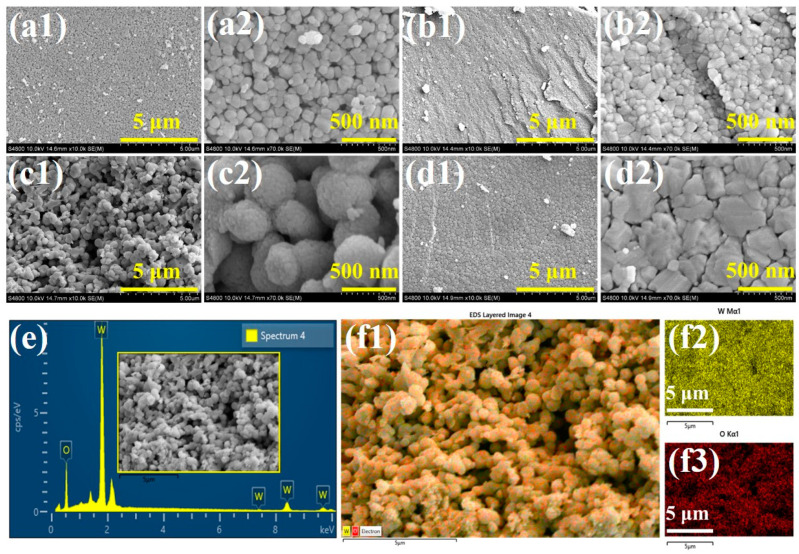
SEM micrograph images of (**a1**,**a2**) WO_3_, (**b1**,**b2**) PEG-WO_3_-1, (**c1**,**c2**) PEG-WO_3_-2, and (**d1**,**d2**) PEG-WO_3_-3. (**e**) Energy-dispersive X-ray spectroscopy analysis and (**f1**–**f3**) elemental mapping data of W and O elements in the PEG-WO_3_-2 electrocatalyst.

The microstructural characteristics and elemental composition of the synthesized electrocatalyst materials were examined using SEM and EDAX. Figure 4(a1,a2,b1,b2,c1,c2,d1,d2) illustrate the morphological variations of WO_3_, PEG-WO_3_-1, PEG-WO_3_-2, and PEG-WO_3_-3 composites, respectively. The SEM analysis of WO_3_ (Figure 4(a1,a2)) reveals the presence of interconnected nanogranular structures with a droplet-like morphology. Upon the incorporation of PEG, a noticeable increase in nanogranular size is observed in PEG-WO_3_-1 (Figure 4(b1,b2)). In the case of PEG-WO_3_-2 (Figure 4(c1,c2)), a porous nanogranular structure with a larger grain size emerges, indicating the impact of PEG concentration on the morphology. With further PEG addition in PEG-WO_3_-3, the structural integrity of WO_3_ is significantly altered, leading to pronounced aggregation as shown in Figure 4(d1,d2). Figure 4e presents the elemental composition analysis performed via EDAX specifically for the PEG-WO_3_-2 composite. The results confirm the presence of tungsten (W) and oxygen (O) with their respective weight percentages determined as 81.76% and 18.24%. To assess the uniformity of elemental distribution, EDAX mapping was conducted as illustrated in Figure 4(f1–f3). The findings demonstrate a high degree of homogeneity in the dispersion of W and O elements throughout the surface of PEG-WO_3_-2. This uniform distribution is expected to enhance the electrocatalytic performance by improving the availability of active sites and facilitating efficient catalytic activity.

**Figure 5:**
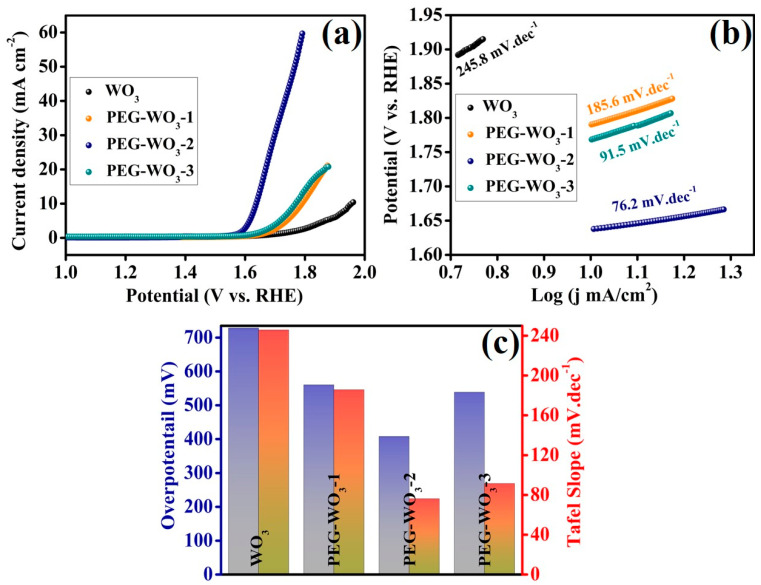
Electrochemical characterizations of electrocatalyst OER performances. (**a**) LSV curves at 5 mV/s scan rate, (**b**) corresponding Tafel slopes, and (**c**) comparison of the OER performance concerning overpotential at 10 mA cm^−2^ and Tafel slope.

Following the structural and microstructural characterization of the synthesized materials, preliminary electrochemical investigations were conducted under alkaline conditions (N_2_-saturated 1 M KOH solution) at ambient temperature to evaluate their electrocatalytic performance. One of the key parameters in assessing the efficiency of electrocatalysts is the overpotential (η) at a specific current density. Initial electrochemical results demonstrated promising OER activity, prompting further in-depth studies to elucidate the electrochemical properties. To determine the OER overpotential of the developed electrocatalysts, LSV measurements were performed. The electrochemical performance of the synthesized materials was examined using a three-electrode system, with the polarization curves of WO_3_ and PEG-modified WO_3_ composites presented in Figure 5. The results indicate that WO_3_ nanogranulars alone exhibit limited OER catalytic activity. However, the incorporation of a surfactant such as PEG into WO_3_ significantly enhances its OER performance. Notably, a slight addition of PEG (PEG-WO_3_-1) resulted in an improved OER response compared to pristine WO_3_. As the PEG content increased, the electrocatalytic behavior exhibited further enhancement as demonstrated in Figure 5a. Among the tested materials, the PEG-WO_3_-2 composite exhibited the highest catalytic activity for OER, surpassing both PEG-WO_3_-1 and PEG-WO_3_-3. The overpotentials recorded at a current density of 10 mA cm^−2^ were 560.3 mV for PEG-WO_3_-1, 407.7 mV for PEG-WO_3_-2, and 538.5 mV for PEG-WO_3_-3, as illustrated in Figure 5a,c. Notably, PEG, a simple linear polymer molecule with a high proton conductivity, which can help protons to leave quickly, may accelerate the O-O bond formation rate and improve the water oxidation kinetics [45,46,47]. The OER performance of PEG-WO_3_-2 alongside other oxides is shown in Table 1. Additionally, Tafel slope analysis was conducted to gain further insights into the OER kinetics and mass transport characteristics (Figure 5b). The calculated Tafel slopes were 245.8 mV dec^−1^ for WO_3,_ 185.6 mV dec^−1^ for PEG-WO_3_-1, 76.2 mV dec^−1^ for PEG-WO_3_-2, and 91.5 mV dec^−1^ for PEG-WO_3_-3. The lower Tafel slope of PEG-WO_3_-2 (76.2 mV dec^−1^) compared to the other composites indicates superior catalytic kinetics and charge transfer efficiency. The relative overpotential and Tafel values for the different catalysts are summarized in Figure 5c. As a result, increasing the PEG content in WO_3_ leads to a significant reduction in overpotential and Tafel slope values, indicating improved electrocatalytic performance. The PEG-WO_3_-2 composition demonstrated the most efficient OER activity among the studied materials, suggesting an optimal balance of PEG concentration for enhanced catalytic behavior.

**Figure 6:**
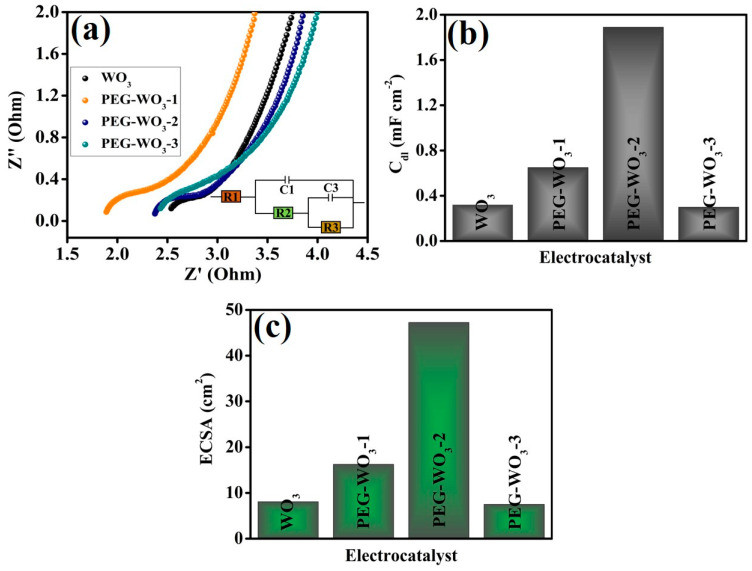
(**a**) EIS spectra, (**b**) C_dl_, and (**c**) ECSA results of all the electrocatalysts.

EIS analyses were conducted to further examine the influence of different electrocatalysts on their electrochemical performance. As illustrated in Figure 6a, EIS measurements were carried out to evaluate interfacial charge transfer resistance (R_ct_) and the reaction kinetics of the OER electrocatalysts. The results indicate that the incorporation of PEG into WO_3_ significantly reduces R_ct,_ thereby enhancing charge transfer efficiency. Among the synthesized composites, PEG-WO_3_-2 exhibited the lowest R_ct_ value, suggesting superior charge transfer capability, which contributes to its enhanced catalytic activity for the OER process. Additionally, ECSA was analyzed to assess the electrochemically active surface area of the catalysts. CV measurements were performed at varying scan rates of 5, 10, 15, 20, and 25 mV s^−1^ under identical experimental conditions. To estimate C_dl_ (Figure 6b) and ECSA (Figure 6c), the values were extracted from the non-Faradaic potential region of the CV curves. The calculated ECSA values depicted in Figure 6c for WO_3_, PEG-WO_3_-1, PEG-WO_3_-2, and PEG-WO_3_-3 are 8.07, 16.25, 47.25, and 7.5 cm^2^. The PEG-WO_3_-2 composite demonstrated the highest ECSA among the tested electrocatalysts. An increased ECSA enhances the interfacial contact area between the electrocatalyst and the electrolyte, thereby improving charge transport and catalytic efficiency [50,51]. The superior ECSA of PEG-WO_3_-2 further supports its enhanced electrocatalytic activity, making it the most effective composition for the OER process.

**Figure 7:**
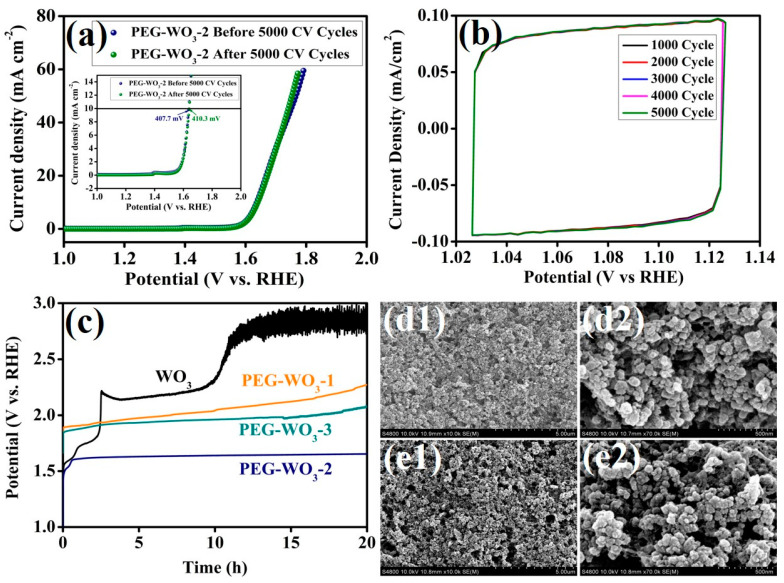
(**a**) LSV curves of PEG-WO_3_-2 before and after 5000 CV cycles (inset overpotential at 10 mA cm^−2^), (**b**) CV stability of PEG-WO_3_-2 over 5000 cycles (1000, 2000, 3000, 4000, and 5000 cycles), (**c**) chronopotentiometry analysis of all the electrocatalyst, and SEM images of PEG-WO_3_-2 electrode on carbon cloth; (**d1**,**d2**) before, and (**e1**,**e2**) after 5000 CV cycles stability test.

Beyond its outstanding electrocatalytic performance, stability is a crucial parameter for evaluating the practical applicability of electrocatalyst materials. To assess the durability of the synthesized electrocatalysts, stability tests were conducted using LSV measurements before and after prolonged CV cycling. As depicted in Figure 7, the stability of the PEG-WO_3_-2 electrocatalyst was examined through LSV analysis following 5000 CV cycles at a scan rate of 50 mV s^−1^ in 1 M KOH. The corresponding LSV polarization curves and 5000 CV (1000, 2000, 3000, 4000, and 5000) cycles are presented in Figure 7a,b. The results indicate that PEG-WO_3_-2 exhibits significant stability as evidenced by the negligible variation in its LSV polarization curves before and after prolonged cycling. Notably, the overpotential at a current density of 10 mA cm^−2^ remained nearly unchanged after 5000 CV cycles with a recorded overpotential of 410.3 mV. The negligible difference in overpotential values before and after cycling confirms the excellent durability of PEG-WO_3_-2. To assess the operational durability of all the electrocatalysts under practical conditions, chronopotentiometry was conducted at a constant current density of 10 mA cm^−2^ for 20 h. As depicted in Figure 7c, Pristine WO_3_ is found to be unstable with increasing potential rapidly. For PEG-WO_3_-1 and PEG-WO_3_-3, a slight increase in potential was observed over time. Significantly, the PEG-WO_3_-2 electrocatalyst displayed exceptional durability, following an initial brief activation phase, the catalyst maintained a remarkably stable operational potential throughout the 20 h test period. The increasing potential trend in the other electrocatalyst, as compared to PEG-WO_3_-2, may be attributed to partial surface site blockage by evolved oxygen bubbles or intermediate species, which can hinder effective charge transfer and contribute to the observed potential rise [52,53]. The before and after 5000 CV cycles stability SEM images of PEG-WO_3_-2 coated on carbon cloth are displayed in Figure 7(d1,d2) and Figure 7(e1,e2). The SEM analysis conducted before and after the stability test reveals that the catalyst retains its surface configuration, with no significant morphological degradation observed. The superior stability of PEG-WO_3_-2 can be attributed to its robust structural integrity and enhanced flexibility, which arise from the strong impact of PEG on WO_3_. These characteristics contribute to the prolonged electrocatalytic performance of the material, advancing it as a promising candidate for practical OER applications.

## 4. Conclusions

In conclusion, this study presents a facile and effective approach for engineering PEG-assisted WO_3_ electrocatalysts via a precipitation method targeting enhanced OER performance in alkaline media. Among the synthesized composites, PEG-WO_3_-2, incorporating 3% PEG, demonstrated the most efficient catalytic activity, exhibiting a low overpotential of 407.7 mV at 10 mA cm^−2^ and a Tafel slope of 76.2 mV dec^−1^ alongside superior long-term stability. This optimal performance is closely correlated with its compositional balance, where the 3% PEG content modulated the crystal structure, increased electrochemically active surface area, and improved charge transfer kinetics. In contrast, both lower (1%) and higher (5%) PEG contents (PEG-WO_3_-1 and PEG-WO_3_-3) resulted in diminished catalytic activity with 560.3 and 538.5 mV overpotential at the same current density due to suboptimal morphology and surface chemistry. These findings underscore the critical role of PEG concentration in tailoring the physicochemical and electrochemical properties of WO_3,_ establishing PEG-WO_3_-2 as the compositionally optimized electrocatalyst. The outcome not only highlights a significant advancement in WO_3_-based catalyst design but also provides valuable insights for the rational development of highly stable and efficient OER electrocatalysts.

## Figures and Tables

**Table 1:** The OER performance of the present work compared with other oxide catalysts.

Electrocatalyst	Electrolyte	Over Potential@10 mA cm^−2^ (mV)	Ref.
Ca-doped CuCoO_2_	1 M KOH	470	[20]
Ni doped CuCoO_2_	1 M KOH	409	[21]
LiCoO_2_	1 M KOH	∼420	[22]
LaFeO_3_	1 M KOH	420	[23]
AgFeO_2_	1 M KOH	400	[48]
CuScO_2_	1 M KOH	490	[49]
PEG-WO_3_-2	1 M KOH	407.7	[Present work]

## Data Availability

The raw data supporting the conclusions of this article will be made available by the authors on request.
